# Ethylene-forming enzyme and bioethylene production

**DOI:** 10.1186/1754-6834-7-33

**Published:** 2014-03-03

**Authors:** Carrie Eckert, Wu Xu, Wei Xiong, Sean Lynch, Justin Ungerer, Ling Tao, Ryan Gill, Pin-Ching Maness, Jianping Yu

**Affiliations:** 1 Biosciences Center, National Renewable Energy Laboratory, 15013 Denver West Parkway, Golden, CO 80401, USA; 2 Department of Chemistry, University of Louisiana at Lafayette, Lafayette, LA 70503, USA; 3 Renewable and Sustainable Energy Institute, University of Colorado Boulder, Boulder, CO 80309, USA

**Keywords:** Ethylene-forming enzyme, Bioethylene, Diversity, Mechanism, Heterologous expression

## Abstract

Worldwide, ethylene is the most produced organic compound. It serves as a building block for a wide variety of plastics, textiles, and chemicals, and a process has been developed for its conversion into liquid transportation fuels. Currently, commercial ethylene production involves steam cracking of fossil fuels, and is the highest CO_2_-emitting process in the chemical industry. Therefore, there is great interest in developing technology for ethylene production from renewable resources including CO_2_ and biomass. Ethylene is produced naturally by plants and some microbes that live with plants. One of the metabolic pathways used by microbes is via an ethylene-forming enzyme (EFE), which uses α-ketoglutarate and arginine as substrates. EFE is a promising biotechnology target because the expression of a single gene is sufficient for ethylene production in the absence of toxic intermediates. Here we present the first comprehensive review and analysis of EFE, including its discovery, sequence diversity, reaction mechanism, predicted involvement in diverse metabolic modes, heterologous expression, and requirements for harvesting of bioethylene. A number of knowledge gaps and factors that limit ethylene productivity are identified, as well as strategies that could guide future research directions.

## Introduction

The rising global demand for petroleum, its restricted supply base, and its deleterious effects on the environment has prompted the development of infrastructure-compatible renewable fuels and chemicals. One potentially renewable feedstock that could have an impact is ethylene. In 2011, the global production capacity of ethylene was 142 million metric tonnes and is forecast to reach 165 million metric tonnes, with an economic impact of US$200 billion per year, by 2015 [1]. Ethylene is the most widely used feedstock in several industries including plastics, textiles, and solvents. In addition, ethylene can also be catalytically polymerized to gasoline-rich hydrocarbons in the C5-C10 range [2,3]. Ethylene is currently produced from steam cracking of fossil fuels or from dehydrogenation of ethane, representing the largest CO_2_-emitting process in the chemical industry. By current state of the art technology, 2 MJ of energy are invested per pound of ethylene made; given the ethylene industry’s massive size, this product alone accounts for 1.5% of United States’ carbon footprint [4]. A renewable route to ethylene production would therefore fulfill an enormous energy and chemical market while helping to preserve the environment.

Ethylene can also be produced biologically. It is a plant hormone that modulates growth and development, and functions in the defense response to abiotic or biotic stress including pathogen attack [5,6]. In plants, ethylene is produced in a two-step reaction from methionine via S-adenosyl-methionine (SAM). SAM is first converted to1-aminocyclopropane-1-carboxylic acid (ACC) by ACC synthase. ACC oxidase then catalyzes the oxidative release of ethylene and cyanide (CN). Although CN is converted to β-cyanoalanine to avoid toxicity in plants, utilization of this pathway for biotechnological ethylene production by other organisms is limited by the need for CN mitigation.

In addition to plants, a variety of microbes including bacteria and fungi also produce ethylene, probably as a causal agent in plant diseases [7]. In *Escherichia coli*, *Cryptococcus albidus*, and a variety of other bacteria, ethylene is spontaneously produced at trace amounts via oxidation of 2-keto-4-methylthiobutyric acid (KMBA), a transaminated derivative of methionine produced in an NADH:Fe(III)EDTA oxidoreductase-mediated reaction that is enhanced under ammonia limitation (C/N = 20) [8,9]. Formation of KMBA is proposed as a means to recover amino nitrogen from methionine, resulting in the spontaneous production of ethylene from KMBA. A third type of ethylene pathway found in *Pseudomonas syringae* and *Penicillium digitatum* utilizes α-ketoglutarate (AKG) and arginine as substrates in a reaction catalyzed by an ethylene-forming enzyme (EFE) [10-14], which will be the focus of this review.

Heterologous expression of a single *efe* gene from *P. syringae* resulted in ethylene production in a number of hosts including *E. coli*[15,16], *Saccharomyces cerevisiae*[17], *Pseudomonas putida*[18], *Trichoderma viride*[19], *Trichoderma reesei*[20], tobacco [21], and cyanobacteria [22-26]. These hosts utilize a variety of carbon sources including lignocellulose and CO_2_, highlighting the various feedstocks that could potentially be used for bioethylene production. In addition, ethylene is not toxic to these organisms, and as a gas it separates easily from cultures. These features compare favorably with other biofuel products such as alcohols or lipids, which tend to be toxic and/or are costly to separate. However, further fundamental and applied studies are needed to bring bioethylene technology to commercial scales. Emerging research topics include a more in-depth understanding of EFE structure and reaction mechanisms, metabolic engineering strategies to improve productivity, and ethylene harvesting technologies. This review aims to provide a summary of the existing literature and to present our own analysis of enzymes and pathways, which together outline a strategy for future research and development of bioethylene production.

### EFE discovery

Ethylene is a hormone that regulates multiple aspects of growth and stress response in plants, and is also a common metabolic product of many fungi and bacteria that live with plants. The common green mold on citrus fruits, *P. digitatum*, was one of the first identified ethylene-producing microbes [10-13]. A cell-free system was prepared from *P. digitatum*[27], and EFE was subsequently purified as a 42-kD protein that required ferrous iron, oxygen, AKG, and arginine for ethylene production [28]. This reaction is in contrast to that in higher plants, which utilize methionine as a precursor in a two-enzyme reaction.

Bacterial production of ethylene was first reported in *Pseudomonas solanacearum* strains, which are involved in early ripening of banana fruits or in wilting of tobacco and tomato [29]. The most efficient microbial ethylene producers include certain pathovars of *P. syringae*. Two of the most studied strains include *P. syringae* pv. *phaseolicola* PK2 (Kudzu strain) and *P. syringae* pv. *glycinea*, which cause halo blight of the vine weed Kudzu and soybean, respectively [7]. Using a cell-free system prepared from the Kudzu strain, it was determined that a 42-kD EFE monomer was required for a reaction utilizing ferrous iron, oxygen, AKG, and arginine, in agreement with EFE studies in the mold *P. digitatum*[14]. Despite the variance of the *P. digitatum* and Kudzu N-terminal sequences [14], the two proteins share overall sequence similarity (see below). The Kudzu *efe* gene was localized to an indigenous plasmid, and when this gene was cloned and expressed in *E. coli*, ethylene production was detected, verifying that expression of a single gene is sufficient for ethylene production in heterologous hosts [15].

### EFE sequence diversity and features

**Figure 1:**
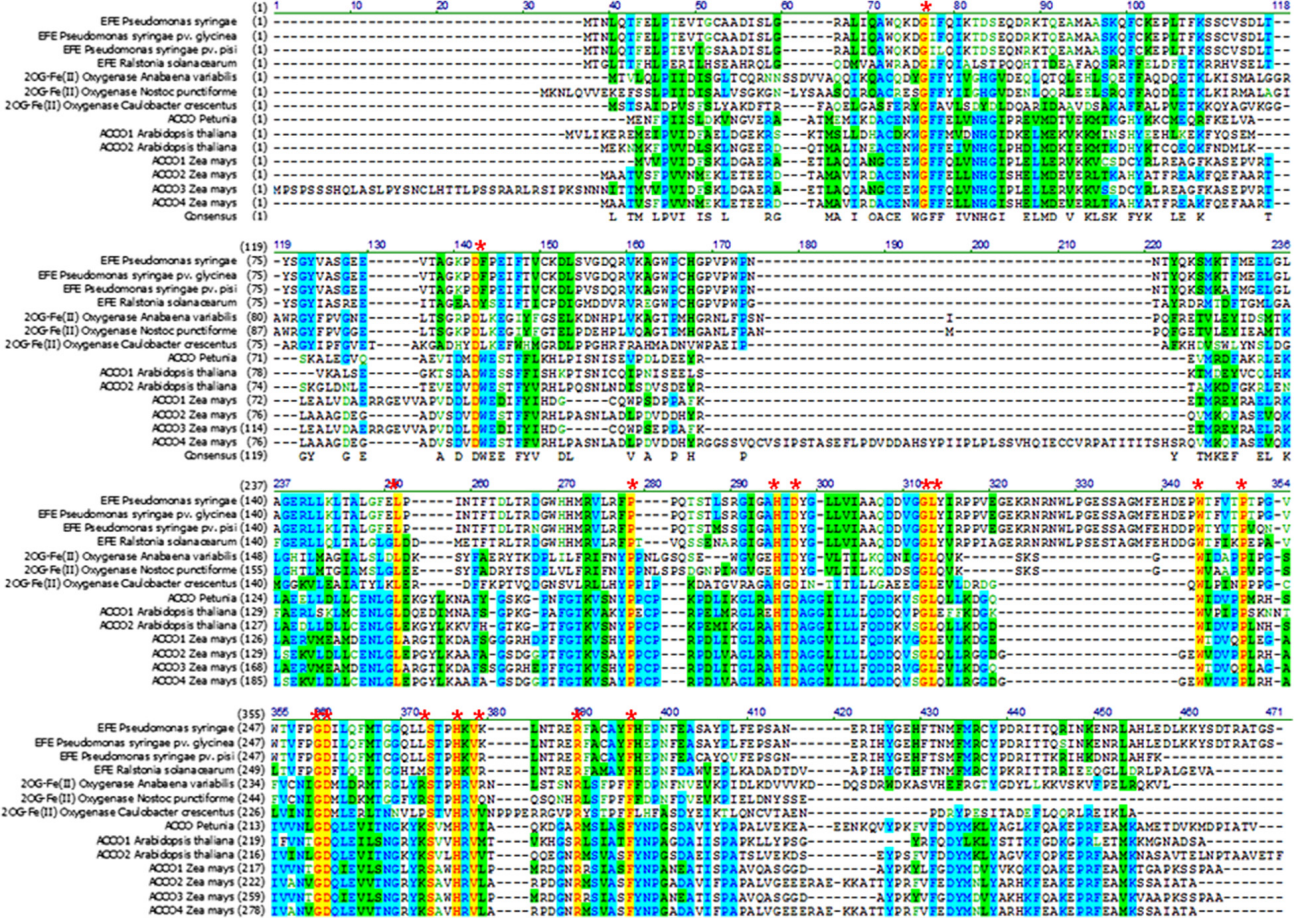
**Phylogenetic study of ethylene**-**forming enzyme (EFE) and EFE-homologous sequences using MEGA 5.2.** The tree could be divided into two large groups and one small group. *Pseudomonas syringae* is the Kudzu strain. EFEs that are heterologously expressed are marked with red stars.

**Figure 2:**
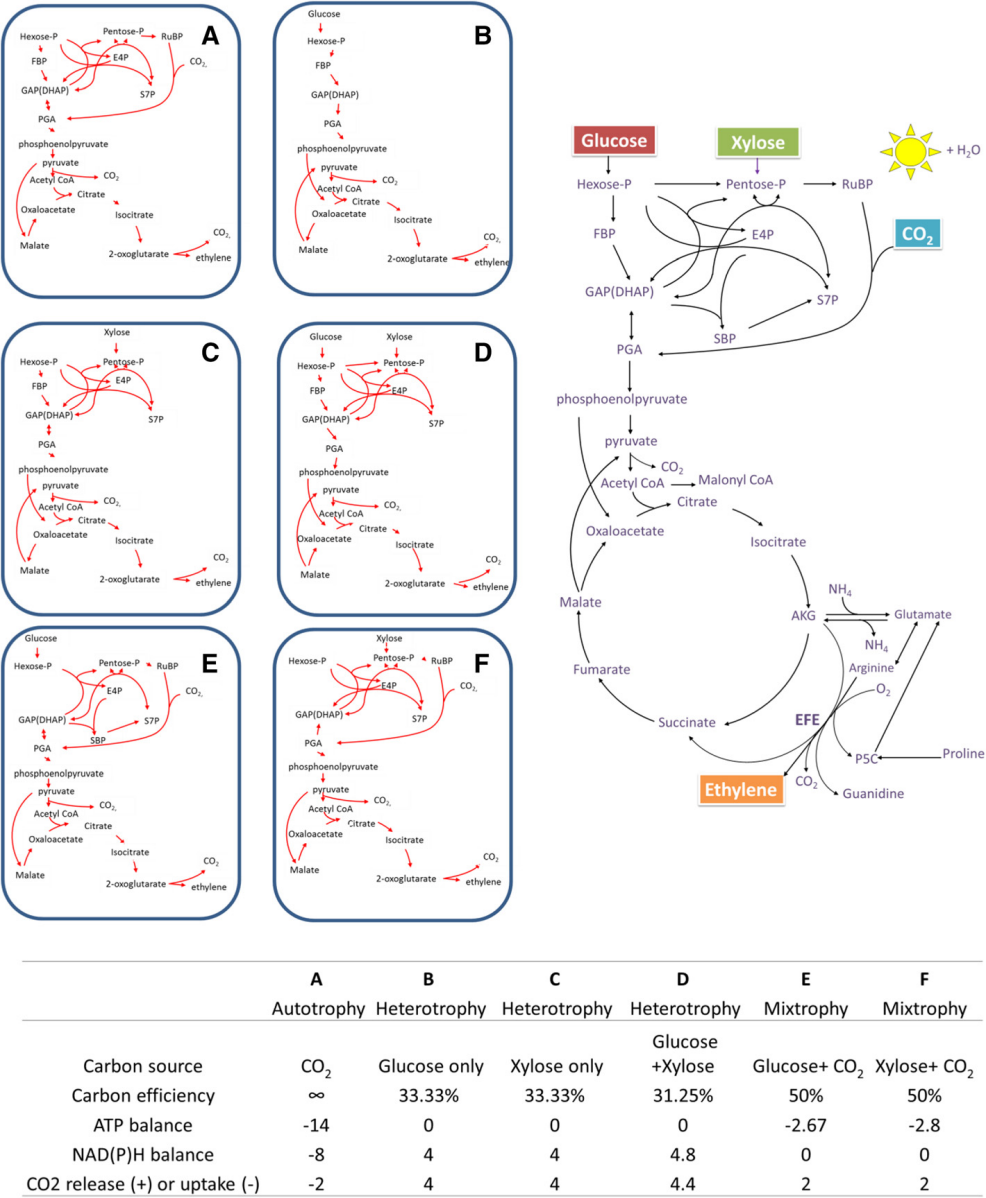
**Sequence alignment of the representative ethylene-forming enzymes (EFEs), 2-oxoglutarate (2OG)-Fe(II) oxygenases and 1-aminocyclopropane-1-carboxylic acid oxidase (ACCO) by ClustalW algorithm.** The red stars show the conserved amino acids. *Pseudomonas syringae* is the Kudzu strain.

To date, sequence diversity of EFEs has not been reviewed, despite the enormous body of available sequencing data. We therefore constructed a phylogenetic tree of EFE based on sequences from the NCBI database with more than 40% identity to that of Kudzu (Figure 1). These sequences can be divided into two major groups and a minor group, with pairwise sequence alignments revealing approximately 25% identity and 65% similarity overall, with the central regions exhibiting the highest conservation (Wu Xu, unpublished data). The identified sequences were annotated as ethylene (succinate)-forming enzyme, 2-oxoglutarate (2OG)-Fe(II) oxygenase, hypothetical protein, 1-aminocyclopropane-1-carboxylic acide oxidase (ACCO), ACC deaminase, and oxidoreductase, suggesting that more functional studies are needed to accurately classify the EFE and EFE-related sequences. As EFEs and ACCOs both catalyze an ethylene formation reaction and belong to the superfamily of 2OG/Fe(II)-dependent hydroxylase enzymes [30], they may share common structural features. To identify conserved amino acids, representative ACCO sequences from the Protein Data Bank (PDB) were compared with Kudzu EFE. The top two enzymes identified are an ACCO from petunia, the only ACCO whose structure has been experimentally determined (PDB ID: 1W9Y; 29.7% similarity and 18.1% identity) [31], and a putative 2OG-Fe(II) oxygenase from *Caulobacter crescentus* (PDB ID: 3OOX; 26.1% similarity and 17.1% identity). ACCOs from *Arabidopsis thaliana* and *Zea mays*, and 2OG-Fe(II) oxygenases from the cyanobacteria *Anabaena variabilis* and *Nostoc punctiforme* were also selected for comparison (Figure 2). The cyanobacterial sequences are of particular interest because of the possibility that their 2OG-Fe(II) oxygenases function as EFE; these cyanobacteria are symbiotic nitrogen-fixing partners with plants, and ethylene production could facilitate the establishment of symbiosis. We found 17 amino acids conserved between Kudzu EFE, ACCOs, and 2OG-Fe(II) oxygenases (Figure 2, highlighted in yellow), suggesting that these residues may play important roles in enzyme structure/function. It has been reported that a majority of the active sites of 2OG/Fe(II)-dependent hydroxylase enzymes contain a single ferrous ion bound in a tridentate ligand arrangement, which is referred to as “a triad of His-Asp/Glu-His.” In the petunia ACCO, His177, Asp179, and His234 form this triad [31]. Based on our sequence alignment, a putative ferrous ion binding site of the Kudzu EFE may consist of three conserved amino acids out of the total of seventeen identified: His189, Asp191, and His268. These three amino acids are physically close in our putative 3D EFE model (Wu Xu, unpublished data). The Kudzu EFE contains 10 His residues, and when site-directed mutagenesis of His to Gln of each was carried out, this resulted in 0 to 60% of activity compared with wild type [32]. Interestingly, mutation of His268 and His189, identified here as possible triad residues, showed enzyme activity of 0% and 1.8%, respectively. Thus, H189, D191, and H268 are the most likely residues for the triad moiety of EFE in the Kudzu strain. Further studies are needed to test this hypothesis.

### EFE reaction mechanism and stoichiometry

Studies of the EFE reaction using cell-free extracts of the fungus *P. digitatum*[27] and the Kudzu strain [33] have led to the following equation for EFE-dependent ethylene production:

### AKG + 3 O_2_ + L-arginine → 2 ethylene + succinate + 7 CO_2_ + guanidine + P5C

Based on the substrate ratio of 3:1 for AKG and arginine and the product ratio of 2:1:1 for ethylene, succinate, and L-delta 1-pyrroline-5-carboxylate (P5C), Fukuda *et al*. proposed a unique dual-circuit mechanism in which EFE catalyzes two different reactions in a 2:1 ratio [33]. In the first (main) reaction (two cycles), arginine remains bound as a cofactor while two AKG are converted to six CO_2_ and two ethylene. In the second (sub-)reaction (one cycle), both AKG and arginine are consumed to yield P5C, guanidine, succinate, and CO_2_. Despite accounting for all of the components added/detected in these *in vitro* studies, the proposed reaction scheme only partially fits the mechanism determined for other related and well-studied enzymes in the superfamily of 2OG/Fe(II)-dependent hydroxylases. The reactions of the latter involve the oxidative decomposition of AKG to CO_2_ and succinate while coupling to the hydroxylation of a co-substrate such as arginine to hydroxyarginine [30]. To address inconsistencies in the EFE reaction mechanism, studies utilizing modern analytical techniques are needed. If the dual-circuit mechanism is correct, is the ethylene-producing catalytic cycle necessarily coupled to the succinate-producing catalytic cycle, or is EFE a promiscuous enzyme capable of catalyzing two distinct reactions, with one being the hydroxylation of arginine and the second the degradation of AKG into CO_2_ and ethylene? If the two reactions are separable, then it may be possible to engineer EFE to produce ethylene without the wasteful generation of side products.

### EFE reaction and cellular carbon flux

Although EFE reaction stoichiometry and the proposed dual-circuit mechanism need further verification, they provide a starting point to analyze the EFE reaction within metabolic networks, providing a systematic overview to enhance understanding and guide engineering approaches.

**Figure 3:**
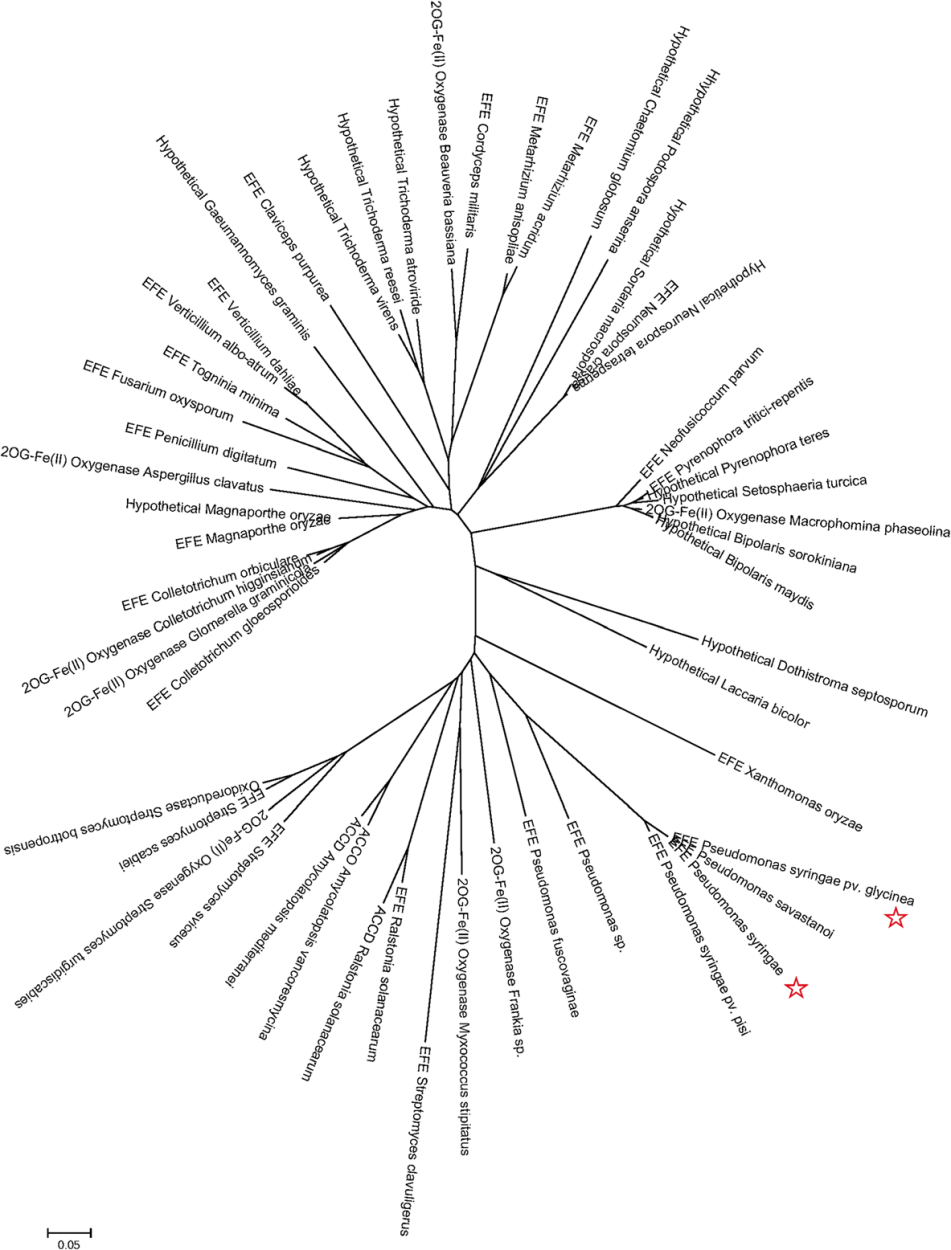
**Various metabolic pathways towards ethylene production.***De novo* synthesis of ethylene by biological systems can be realized by using either organic or inorganic substrates (for example, glucose, xylose and CO_2_) in a global metabolic network (top right). Detailed in panels are the metabolic routes applying various combinations of substrates: **(A)** CO_2_ only (autotrophic), **(B)** Glucose only (heterotrophic), **(C)** xylose only (heterotrophic), **(D)** glucose + xylose (heterotrophic), **(E)** glucose + CO_2_ (mixotrophic), and **(F)** xylose + CO_2_ (mixotrophic). Corresponding carbon efficiency or yield (carbon stored in ethylene/carbon uptake), CO_2_ release/uptake, and cofactor balances in each panel are normalized to the formation of 1 mole of ethylene, and presented in the table (bottom right). Note that positive cofactor balances represent net production, while negative ATP or NADPH balances require cofactor supply from elsewhere (for example photosynthetic light reactions). The stoichiometries are calculated with computational analysis through determination of elementary modes for a given reaction system [34,35]. For computational analysis, all possible routes for conversion of organic/inorganic carbons to ethylene were considered. The reaction for ethylene production in panels **(A-F)** is defined as: α-ketoglutarate = ethylene + 3 CO_2_. Side reaction of ELE is not taken into account, because of the controversial and uncertain stoichiometry. CO_2_, carbon dioxide; DHAP, dihydroxyacetone phosphate; E4P, erythrose-4-phosphate; FBP, Fructose 1,6-bisphosphate; GAP, glyceraldehyde-3-phosphate; Hexose-P, hexose 6-phosphate; Pentose-P, pentose 5-phosphate; PGA, phosphoglycerate; RuBP, ribulose 1,5-bisphosphate; S7P, sedoheptulose-7-phosphate; SBP, sedoheptulose 1,7-bisphosphate.

Efficient ethylene biosynthesis should involve metabolic pathways for which the molar yield (product:substrate molar ratio) is maximal for ethylene and minimal for byproducts and energy/cofactor consumption. Elementary node analysis [34,35] was applied in this study, and Figure 3 shows theoretical yields and cofactor costs for ethylene conversion from common substrates, including CO_2_ (via photosynthesis), glucose, and xylose, all possible feedstocks for the heterologous EFE-expressing hosts outlined in the next section. Figure 3A represents the classic photoautotrophic pathway for the conversion of CO_2_ to ethylene via the Calvin Benson Bassham cycle, and shows net carbon uptake and consumption of ATP and reductant (generated by photosynthetic light reactions). Figure 3B represents glycolysis from hexose to ethylene, resulting in 33.33% carbon yield and net reducing equivalents via the NAD(P)^+^-dependent activities of glyceraldehyde 3-phosphate dehydrogenase, pyruvate dehydrogenase, and isocitrate dehydrogenase (ICD). To reach the maximum theoretical yield, six carbons of a glucose molecule are split into two three-carbon units via glycolysis, one carbon is gained from the C4 route, and a total of five carbons are lost via pyruvate dehydrogenase (one carbon), ICD (one carbon), and EFE (three carbons), respectively. Utilization of xylose (Figure 3C) or mixed sugars (xylose plus glucose, Figure 3D) bypasses glycolytic flux via the pentose-phosphate pathway, resulting in a similar ratio of reductant production and carbon yield as seen for glycolysis (Figure 3B). The routes outlined in Figure 3E and Figure 3F utilize the CO_2_-fixing enzyme ribulose-1,5-bisphosphate carboxylase/oxygenase (Rubisco) as a non-oxidative shunt, reducing total carbon release in xylose-feeding or glucose-feeding systems, respectively. The additional one-pass flux through Rubisco increases carbon yield to 50%, but there is a trade-off at the cost of increased cofactor requirements. Comparison of these metabolic routes reveals that photobiological conversion of CO_2_ to ethylene represents a carbon-negative process with a high demand for photosynthetically derived ATP and reducing equivalents, while heterotrophic ethylene production from hexose/pentose has no net cofactor input but still results in carbon loss. Therefore, strategies to recycle/avoid lost carbon must be considered. Recruiting both photosynthetic (could be more than one pass) and sugar-utilizing pathways in a mixotrophic mode should result in a higher theoretical yield and lower cofactor requirement simultaneously. In addition, carbon yield may be improved with pathways that bypass pyruvate dehydrogenase, or with a more efficient EFE (previous section).

Besides identifying the optimal pathways, metabolic bottlenecks and competing pathways can be revealed by systems biology approaches such as flux balance analysis (FBA) and metabolic flux analysis (MFA). MFA can help identify rate-limiting factors that control flux through EFE, and can be used to measure EFE and competing fluxes *in vivo*. An analysis of metabolism towards ethylene formation was performed in genetically engineered *S. cerevisiae* using FBA [36], which used linear optimization to determine the steady-state reaction flux distribution in a mathematic network by maximizing ethylene production as an objective function [37]. In that study, either S-adenosylmethionine-dependent (via ACC) or AKG-dependent EFE were added into the reaction network, and optimized for maximal ethylene formation. The optimal ethylene yields calculated for the two systems were both in the range of 7–8 moles of ethylene/10 moles of glucose, or a carbon yield of 23.33 to 26.67% [36]; the maximal theoretical yield would be 33.33% when only minimal enzyme sets for ethylene production are considered (see above). Potential strategies to increase ethylene formation were also analyzed. The authors suggested that supplementation of exogenous proline, using a solely NAD-coupled glutamate dehydrogenase (catalyzes glutamate to AKG), and use of glutamate as the nitrogen source could increase ethylene formation. The study also indicated that computational results are close to experimentally observed values when additional constraints such as a constraint on respiratory capacity (for example, limiting O_2_ or not) are defined. Future work to identify the most efficient routes to ethylene production should include isotope labeling such as ^13^C MFA [38,39] to allow for profiling of actual flux maps to complement the *in silico* modeling approaches.

### Heterologous expression of EFE and ethylene production

Metabolic engineering to improve ethylene production and better understanding of metabolic flux to ethylene are necessary to realize bioethylene production on an industrial scale. As only one gene (*efe*) is necessary for ethylene production from common metabolites, it is of great interest to study the heterologous expression of EFE in organisms that can utilize a variety of feedstocks. Collectively, ethylene production has been successfully demonstrated in engineered microbes utilizing diverse renewable resources such as sunlight, cellulose, or biomass-derived glucose.

#### E. coli and S. cerevisiae

The earliest efforts at heterologous expression of EFE involved cloning of the Kudzu *efe* gene (with its native promoter) into a high-copy pUC19 vector and expressing it in *E. coli*, resulting in measurable ethylene production (Table 1) [15], and revealing that *efe* is sufficient for ethylene production in a foreign host. In a follow-up study, increased EFE expression from a *lac* promoter on a high-copy pUC18 vector, or from a *tac* promoter on a medium-copy pBR322 vector, led to much higher activities when cultures were grown at 25°C (Table 1, [16]). Alternatively, when cultures were grown at 37°C, very low activities were detected at all stages, in agreement with the short half-life (3.3 minutes) of the EFE protein at 37°C. Furthermore, raising the temperature from 25°C to 37°C led to increased localization of the protein in inclusion bodies. Interestingly, a short peptide (15 amino acids) from LacZ at the N-terminus in the strain overexpressing EFE (from the *lac* promoter) led to decreased localization of EFE in inclusion bodies, but activity at 37°C remained low [16], suggesting that other factors probably also affect EFE activity and stability.

**Table 1:** **Ethylene productivities in EFE**-**expressing microbes**

**Host**	**Native, vector, or integrated EFE expression**	**Promoter**	**Temperature, °C**	**Feedstock**	**Rate of ethylene production. (μmol/gCDW/h)**	**Ref.**
*Pseudomonas syringae* (Kudzu)	Native	Kudzu	30	LB + 0.5% glucose	39.0	18
*P. syringae* (Kudzu)	Native + vector (RS1010)	Kudzu, npt	30	LB + 0.5% glucose	312.0	18
*Escherichia coli* (JM109)	Vector (pUC19)	Kudzu	37	Modified LB	10.9	15
*E. coli* (DH5α)	Vector (pUC18)	lac	25	LB	625.0	16
*E. coli* (JM109)	Vector (pBR322)	tac	25	LB	412.9	16
*E. coli* (JM109)	Vector (RS1010)	npt	30	LB + 0.5% glucose	55.2	18
*E. coli* (DH5α)	Vector (RS1010)	lac/trc	30	LB	22.8	26
*E. coli* (MG155)	Vector (pBR322)	psbA	30	M9 + 1% glucose	30.0	UP
*Saccharomyces cerevisiae* (batch)	Vector (pYX212)	tpi	30	YNB + 1% glucose + glutamate	21.4	17
*S. cerevisiae* (chemostat)	Vector (pYX212)	tpi	30	CBS + 1% glucose + (NH_4_)_2_SO_4_	1083.8	42
*S. cerevisiae* (chemostat)	Vector (pYX212)	tpi	30	CBS + 1% glucose + glutamate	1151.5	41
*S. cerevisiae* (chemostat)	Vector (pYX212)	tpi	30	CBS + 1% glucose + glutamate + arginine	492.0	42
*Synechococcus* 7942	Vector (pUC303)	psbA1	25	BG11	84.8	23
*Synechococcus* 7942	Integrated (psbAI)	psbA1	28	BG11	80.5	24
*Synechocystis* 6803	Vector (RS1010)	lac/trc	30	BG11	26.0	26
*Synechocystis* 6803	Integrated (slr0168)	psbA	30	BG11	111.6	UP
*Trichoderma viride*	Integrated (random)	cbhI	30	MM + 2% cellulose +0.2% peptone	0.093	19
*Trichoderma reesei*	Integrated (random)	pgk	30	MM + 2% wheat straw	0.716	20
*Pseudomonas putida*	Vector (RS1010)	npt	30	LB + 0.5% glucose	1050.0	18
*P. putida*	Integrated (five 16S rDNA sites) + vector (pBBR1MCS2)	rrn	28	LB	2859.2	48

For comparison, reported rates have been converted to μmol**/**gCDW/h.

CDW, cell dry weight; EFE, ethylene-forming enzyme.

In addition to levels of active EFE, substrate availability may also limit ethylene production in heterologous expression systems. When Kudzu EFE was expressed under the constitutive *npt* promoter from a low-copy plasmid (RS1010) in *E. coli*, *P. putida*, and *P. syringae* (containing native gene + plasmid-based overexpression), *P. putida* exhibited the highest maximal rates of ethylene production (Table 1) [18]. Maximal activity in all three overexpression systems occurred early in growth and fell off rapidly, consistent with previous observations in *E. coli*[16]. Additionally, *in vivo* EFE activities (intracellular substrates only) of cultures sampled at time points with maximal production rates were compared with those *in vitro* (exogenously added substrates at saturating levels). These comparisons revealed that although WT *P. syringae* had similar EFE activities *in vivo* and *in vitro*, the *in vitro* activities from the *E. coli, P. putida*, and the *P. syringae* overexpressing strain, were, respectively 5-fold, 20-fold, and 40-fold higher than those seen *in vivo* suggesting that substrate availability limits *in vivo* activity [18]. Zhang *et al*. recently reported that intracellular levels of AKG reached their highest levels in early growth [40], consistent with the above observation that AKG levels may be limiting.

Pirkov *et al*. observed that in *S. cerevisiae*, ethylene production nearly tripled when the nitrogen source in minimal media (1.0% glucose) was changed from ammonium to glutamate in batch cultures when the Kudzu *efe* gene was expressed by a strong, constitutive *tpi* promoter on a multicopy 2 μ plasmid (Table 1) [17], in agreement with an *in silico* production model (see previous section) [36]. This model also revealed that experimentally measured ethylene yields were consistent with the yields predicted under limited respiration (Table 1) [36], suggesting that O_2_ availability is necessary for maximal ethylene production. In a more recent study, ethylene production was further analyzed in a chemostat with increased O_2_, leading to improvement in ethylene production by more than 53-fold over that seen in batch cultures (Table 1) [41]. When the nitrogen source was changed from ammonium to glutamate, growth was improved, but no change in specific ethylene productivity was seen, suggesting that the improvement observed with glutamate addition to batch cultures was due to cell growth and not increased EFE productivity (Table 1) [41]. Furthermore, addition of the EFE substrate arginine actually reduced ethylene productivity by over half (Table 1) [41]. The authors postulated that addition of arginine may result in a “push” towards the succinate-forming sub-reaction proposed by the dual-circuit mechanism [41]. Together, these studies highlight that beyond strategies to improve EFE stability, further analysis of substrate enrichment and increased O_2_ availability are necessary to maximize ethylene production.

#### Cyanobacteria

To link ethylene production to photosynthetic CO_2_ fixation, the *efe* gene from the Kudzu strain was heterologously expressed in cyanobacteria in a number of studies. In *Synechococcus elongatus sp*. PCC 7942, vector-based expression of EFE was first explored from a low-copy pUC303 vector [22,23]. Interestingly, unlike that seen for heterologous expression in *E. coli*, the *in vivo* and *in vitro* activities were comparable, suggesting that substrates for the EFE reaction are not limiting in *Synechococcus*[23]. When plasmid-based expression of EFE was analyzed using a variety of promoters, a native *psbA1* promoter exhibited the highest activity (Table 1) [23], although vectors containing more than 100-bp homology to this native *psbA1* promoter region were unstable. Plasmid instability was correlated with slow growth, bleaching, and a decreased CO_2_ to ethylene partition rate compared with strains containing vectors with no/low native sequence (100 bp or fewer), and lower rates of ethylene production [23]. The authors postulated that decreased fitness could be a result of plasmid loss (loss of antibiotic resistance in the presence of antibiotic) and/or metabolic stress linked to EFE activity, as the addition of ethylene to cultures did not affect growth [23].

To address such instability issues, the *efe* gene was integrated at the *psbA1* locus in *S. elongatus* sp. PCC 7942 [24,42,43]. When a kanamycin resistance gene was additionally integrated behind the *efe* gene, ethylene production was stable over 30 generations [42,43]. A markerless insertion of *efe* at the same locus exhibited rates of ethylene production that were four times higher than in strains containing the integrated kanamycin resistance gene [24], exhibiting rates similar to those in the best plasmid-based expression strains (Table 1) [23,24]. These markerless integration strains similarly exhibited defective growth and metabolic stress when active EFE was expressed [23,24]. The authors suggested that with increased ethylene production, levels of AKG become limiting, hence shifting glutamate to AKG instead of to bilin production [44], leading to cell bleaching and slowed growth rates. It is unknown whether increased availability of AKG (as well as arginine) would increase ethylene production and/or rescue the growth defects in these strains.

Expression of Kudzu EFE from the low-copy RSF1010 plasmid was compared in *E. coli* and the unicellular cyanobacterium *Synechocystis* sp. PCC6803 [26]. Unlike plasmid-based expression in *Synechococcus*, expression of EFE from this vector did not lead to plasmid instability. The highest rates of production were achieved for both *E. coli* and *Synechocystis* when the *trc* or *lacO*-*1* promoters were utilized, although expression in *Synechocystis* was independent of IPTG addition (Table 1[26]).

The *efe* gene from the Kudzu strain has also been codon-optimized and integrated into the genome of *Synechocystis*[25]. Stable expression of the EFE was achieved and optimized [25] using a constitutive, high-level pea plant chloroplast *psbA* promoter (σ^70^ consensus [45]) to drive its expression when integrated at the *slr0168* neutral-site locus [46]. Current work to increase EFE expression levels has led to even higher rates of ethylene production (Table 1) (Jianping Yu, unpublished). It is currently unknown whether the EFE sequestration or stability issues discussed above similarly affect ethylene production in this cyanobacterium.

#### Cellulolytic fungi and microbes that utilize diverse feedstocks

With many fungi exhibiting strong cellulolytic activity, expression of EFE in fungal hosts provides a promising route for ethylene production from renewable biomass. Tao *et al*. reported the successful heterologous expression of an integrated *P. syringae* pv. *glycinea efe* gene driven by the strong *cbhI* promoter in *T. viride*[19]. Maximal production rates were observed when 2.0% cellulose and 0.2% peptone were used as carbon sources, with the addition of peptone having the most significant impact on production (Table 1) [19]. Another cellulolytic fungus, *T. reesei*, was also analyzed as a host for expression of *efe* from *P. syringae* pv. *glycinea*[20]. The *efe* gene was randomly integrated into the genome and expressed by a variety of promoters, and resultant strains were screened for the highest rates of production. A strain expressing *efe* from the *pgk* promoter of *T. reesei* demonstrated the highest activity (Table 1) [20].

*P. putida* is a Gram-negative soil bacterium with a diverse metabolism that has potential for the production of a variety of compounds using various waste streams as feedstock. Based on the high ethylene production rates exhibited by *P. putida* expressing Kudzu EFE from a plasmid (see above), Wang *et al*. designed a vector to integrate multiple copies of the *efe* gene (from *P. syringae* pv. *glycinea*) into the 16S rDNA sites of *P. putida*[47]. The use of this construct led to the integration of 3–5 copies of the *efe* gene, with expression driven by the native *rrn* promoter. Ethylene production rates increased with increasing copy number, with the highest rate achieved in the strain containing five integrated copies in addition to expression from a medium-copy, broad-host range plasmid. This strategy ultimately increased ethylene production and glucose-to-ethylene conversion to the highest rates by native and recombinant organisms reported to date (Table 1) [47].

### Harvesting of biologically produced ethylene

One important consideration for the biological production of ethylene is harvesting. In the petrochemical industry, ethylene is typically harvested from a gaseous mixture via cryogenic distillation [48], which is energy-intensive but is capable of harvesting multiple gaseous products in the mixture. Other methods include solvent extraction, pressure swing adsorption using zeolites, and membrane separation. Special consideration must be implemented in the harvesting of biologically produced ethylene, depending on the gas composition in the mixture. It is expected that besides ethylene, there may also be CO_2_, water vapor, N_2_, and O_2_ present in the biologically derived gaseous stream. When O_2_ is co-produced with ethylene in a photosynthetic system, there is an important safety issue regarding the flammability of ethylene in the presence of O_2_ (2.7 to 36% v/v [49]). Therefore, engineering designs must be included to mitigate this risk. Biologically produced ethylene is expected to be free of metals and other contaminants commonly found in fossil-derived ethylene stream, and therefore may become a preferred feedstock for high-purity chemicals and clean fuels.

### Future research directions

The development of bioethylene technologies is in its infancy. In order to confer a major impact in displacing fossil-derived feedstocks, advances in many research areas are needed to improve ethylene production strains, cultivation systems, and harvesting technologies. Work is ongoing to conduct a technoeconomic analysis of bioethylene production in a cyanobacterial system. Its outcomes will provide parameters to guide future directions in research and development.

As discussed above, fundamental knowledge of the structure, function, and reaction mechanism of EFE is currently lacking. The analysis of EFE and its related sequences and structures to identify conserved regions and a putative enzyme active site will aid research to evaluate these features and the proposed dual-circuit catalytic mechanism. A crystal structure of EFE will additionally enhance our understanding of this enzyme and guide protein engineering towards increased carbon yield and thermal stability. Furthermore, accurate reaction stoichiometry coupled with carbon flux analysis will guide metabolic pathway engineering to construct more efficient production route(s).

The advent of synthetic biology will also accelerate strain development by optimizing the design of pathways for high-yield ethylene production. To realize the full potential of a synthetic biology-based engineering approach, high-throughput screening/selection tools are needed to monitor levels of ethylene and its precursors. Currently, genetically encoded, sensor-based screens have been developed for AKG [40] and arginine [50], and a direct ethylene sensor could potentially be constructed based on the ethylene receptor in plants and cyanobacteria [51].

Lastly, for scaled-up production, inexpensive bioreactors must be developed with enhanced O_2_ mass transfer for non-photosynthetic systems, light delivery for photosynthetic systems, and associated harvesting systems tailored to a biologically derived gas stream.

## Conclusions

During the course of evolution, microbes have developed multiple ethylene-producing pathways to take advantage of ethylene-responsive mechanisms in plants and facilitate the successful invasion of plant tissue. The outcome may be beneficial only to the microbes in the case of pathogenesis, or it may be mutually beneficial in the possible case of symbiosis. Nevertheless, the underlying mechanism governing EFE catalysis remains as an emerging research topic for the production of bioethylene. With the advent of synthetic biology tools and advanced analytical capabilities, robust ethylene production via EFE can be exploited in heterologous systems for production of this versatile feedstock from diverse renewable resources such as biomass, sunlight, and CO_2_. The successful outcome will reduce our dependence on fossil fuels, and provide a viable feedstock for bio-based chemicals and fuels.

## Abbreviations

2OG: 2-oxoglutarate; AKG: alpha-ketoglutarate; ACC: 1-aminocyclopropane-1-carboxylic acid; CBB cycle: Calvin Benson Bassham cycle; EFE: ethylene-forming enzyme; FBA: flux balance analysis; KMBA: 2-keto-4-methylthiobutyric acid; MFA: metabolic flux analysis; P5C: L-delta-1-pyrroline-5-carboxylate; Rubisco: ribulose-1,5-bisphosphate carboxylase/oxygenase; SAM: S-adenosyl-methionine.

## Competing interests

The authors declare they have no competing interest in this work.

## Authors’ contributions

CE, RG, PCM and JY conceived the project and edited the manuscript. All authors analyzed and interpreted data and drafted various parts. In particular, PCM drafted the introduction; JY drafted “EFE discovery and future research directions”; WXu drafted “EFE diversity and features”; JU and JY drafted “EFE reaction mechanism; WXiong drafted “EFE and metabolic modes”; CE and SL drafted “EFE heterologous expression and ethylene production”; LT drafted “ethylene harvesting”; RG drafted the conclusion. All authors read and approved the final manuscript.
